# Tablet-Based Well-Being Check for the Elderly: Development and Evaluation of Usability and Acceptability

**DOI:** 10.2196/humanfactors.7240

**Published:** 2017-05-12

**Authors:** Pradeep Ray, Junhua Li, Arni Ariani, Vasvi Kapadia

**Affiliations:** ^1^ Centre For Entrepreneurship University of Michigan Joint Institute Shanghai Jiao Tong University Shanghai China; ^2^ WHO Collaborating Centre on eHealth School of Public Health and Community Medicine UNSW (University of New South Wales) Sydney Australia; ^3^ Hammondcare Sydney Australia

**Keywords:** agile methodology, elderly people, information and communications technology application, silvercare model, tablet-based well-being system

## Abstract

**Background:**

Many elderly people prefer to live at home independently. One of the major concerns raised by the family members is the safety and well-being of their elderly family members when living independently in a home environment. To address this issue, assistive technology solutions have been available in the market. Despite their availability and proliferation, these types of solutions are not popular with the elderly due to their intrusive nature, privacy-related issues, social stigma, and fear of losing human interaction. This study shares the experience in the development of a digital photo frame system that helps family members to check the well-being of the elderly, exploiting their desire to remain socially connected.

**Objectives:**

The aim of this study was to iteratively design, implement, and assess the usability, user friendliness, and acceptability of a tablet-based system to check the well-being of the elderly.

**Methods:**

Our study methodology comprises three separate stages: initial system development, contextual assessment, and comparative case study evaluation.

**Results:**

In the first stage, requirements were elicited from the elderly to design a well-being check prototype. In the second stage, areas for improvements (eg, privacy features) were identified. Also, additional features (such as medication prompts or food reminders) were suggested to help aged and health care service providers with effective but subtle monitoring of the elderly. These would lower their operating cost by reducing visits by care providers to the homes of the elderly. In the third stage, the results highlighted the difference (between users in India and Australia) in the levels of familiarity of the elderly with this technology. Some elderly participants at the Kalyani Institute for Study, Planning and Action for Rural Change, India latched onto this technology quickly while a few refused to use the system. However, in all cases, the support of family members was crucial for their willingness to use the technology.

**Conclusions:**

This project has three major outcomes. First, a picture frame prototype was tested with the elderly to leverage the benefits of social communication. Second, the project helped us test and implement the “Silvercare” model for supporting the elderly through young retired people residing in the area. Finally, the project helped formalize the agile three-stage design methodology to develop information technology solutions for the elderly. Also, the project contributed to an ongoing European Union Project called Victoryahome, which involves more than 50 sites across 5 countries (Norway, Sweden, Netherlands, Portugal, and Australia) to assess the use of telepresence robots, wearable fall detectors, and medication dispensers for the elderly living independently.

## Introduction

### Background

Between 2000 and 2050, the population aged above 65 years will double from the current 8%-16% in the world [1]. In Australia, the number of senior citizens aged over 65 years is projected to nearly double its population from 13.5% of a population in 2010 to 22.7% by 2050 [2].

In 2006, 2.74 million people in Australia were aged 65 and over and around 29% of them (0.78 million) were living alone and independently [3]. However, there have been risks associated with greater numbers of the elderly living alone at home, such as a higher risk of falls [4]. In this case, the extended family members are often worried about the well-being of elderly family members [5].

There is a growing demand for well-being monitoring technologies such as fall detection, remote health monitoring, smart home solutions, and video surveillance, which would provide the elderly with a sense of security and independence [6]. Researchers believe that these well-being technologies have their potential to reduce the number of visits to clinics and hospitals [7].

Existing technological solutions for aged care are often designed from a technical perspective and do not appropriately address the needs and/or preferences of the elderly [8]. Hence there is a need to involve the elderly in the development process of the well-being technology [9]. Also, a closer look at the gerontology literature and research is required to understand the values and attitudes of the elderly toward technology.

Some studies have revealed that the elderly mostly adopt and use technology with an element of human interaction [9-11].

A recent study concluded that there were significant changes in the elderly’s attitude toward information and communications technology (ICT) [12-14]. Most participants admitted that the Internet helped them to maintain constant communication with family members and friends [12]. The Internet has also been used by the elderly to pay bills, access Web-based banking services, and search for information on their health conditions [12,14]. In keeping with the trend, technology vendors are developing ICT systems that can bridge and enhance social interactions between the elderly and their family members [15].

One of the examples is an interactive, digital photo frame that enables both the elderly and their family to share pictures, memorable moments, and their activities [5]. The solution is Web-based and runs on a wide range of devices like iPad and Android tablets. It can enhance social interaction and check the well-being of the elderly. Its development adopted an agile methodology and involved multiple end users and stakeholders.

This paper is organized as follows. The paper starts with a literature review of the existing well-being technologies for the elderly. This is followed by a discussion of the needs for a methodology to develop information technology solutions for the elderly, incorporating participatory design involving the elderly at several stages. This is followed by a description of our three-stage methodology for the design and assessment of a tablet-based well-being system for the elderly. The paper concludes with a short discussion.

### The Existing Well-Being Technologies for the Elderly

Most technology products available for the elderly in the market are designed to enhance the functional abilities of the elderly, such as assistive robots [16,17], digital photo frames [18,19], telehealth [20,21], smart homes [22,23], video games [24,25], and video surveillance for fall detection [26,27]. During their design and development, there has been minimal or no involvement of the elderly to understand their needs and requirements [28]. Some research findings have demonstrated the importance of addressing human factors in designing ICT products to attain the positive impact on the elderly’s well-being [29,30]. The following review below discusses the existing well-being technology solutions and main issues in their adoption.

There are a growing number of studies on animal robots to provide emotional support and treatment to the elderly [31,32]. Two of the most prominent examples are a baby seal robot called “Paro” [15] and a robotic dog called “AIBO” [32,33]. Some studies found that the availability of “Paro” could have a beneficial effect on the quality of the elderly’s life (both physiologically and socially) [32,33]. In a study by Kidd et al [33], it was found that the elderly formed a special attachment to Paro. As therapy aids, AIBO facilitated effective communication between the elderly, their family, and nursing homes, and improved the quality of the elderly’s life [34].

Some of these assistive robots were designed to establish engagement with and provide enjoyable experience to the users; nevertheless, a deeper emotional bond may cause unwanted situations [35]. Findings from Tapus et al [36] showed that the elderly with Alzheimer disease felt emptiness and loss when the robot was removed from their side.

A study conducted by Mynatt et al [5] has revealed that digital family portraits with a qualitative visualization of the family members’ life allow them to remain emotionally connected. This study has also found that that sharing day events by sending photos and drawings on display screens would be greatly appreciated to support communication between the elderly and their geographically distant family members [5].

Telehealth can be used to monitor the health status of people with chronic diseases (such as cardiovascular diseases, diabetes, and obesity) [37]. For example, Holter monitors and automatic blood pressure (BP) cuffs are routinely used to measure heart rhythm/rate and BP, respectively [37]. Some studies have suggested that the elderly may object and refuse to adopt wearable sensors due to a number of reasons such as the wearable sensor being uncomfortable to wear and being viewed as a stigmatizing symbol of their frailty or age [21,38].

Wireless sensor monitoring is a rapidly emerging area that supports the elderly living independently [22]. It is currently being developed to help elderly people achieve greater levels of safety and independence [22]. Care systems such as smart homes are being equipped with multiple sensors that can interact with other sensors worn by the elderly [23].

A study by Zhang et al [39] used ambient sensors (motion infrared detectors and pressure mat sensors) to detect a fall at the night time in a one-person household. The limitation of using ambient sensors is that it can only monitor 1 person during the night time and it is not suitable for multiple occupancy households (ie, residential aged care or nursing home facilities).

Recently, a number of studies have been conducted to investigate therapeutic effects of video games for the elderly [24,25]. A study by Lee and Shin [24] used video games (ie, PlayStation 2, Sony, Tokyo, Japan) to improve their balance, gait, falls efficacy, and strength.

In general, two common causes of problems during gaming sessions are aged-related physical and mental changes and lack of familiarity with video games [25]. Ijsselsteijn et al [40] have accordingly made recommendations for the design of video games for the inexperienced elderly: (1) the first-level games, which ensure that the elderly are already able to master the basic skills needed before continuing with the next levels of games, and (2) a feature that provides continual performance feedback at each level of the games.

In a study by Fleck and Straßer [26], multiple cameras were placed around a health facility to determine the occurrence of a fall, and Google Earth was used to display the floor plan of the health care facility [27]. In the case of a fall, the system would immediately send an emergency alert and automatically share the world-coordinate location of the faller [26].

Video surveillance has been chosen over other sensor technologies due to a number of reasons. The price of video cameras is decreasing rapidly [41]; the system is able to detect multiple events simultaneously; and the user is not required to wear any devices all the time [42]. Since the surveillance system records all activities inside the house, security and privacy-related issues are extremely crucial to be considered [43,44].

Although there have been attempts to design and evaluate the above types of technologies for the elderly, there is no integrated, systematic methodology for the design and evaluation of information technology solutions for the elderly. We now discuss the field from such a methodology perspective.

## Methods

### Study Design

The main barriers to the deployment of assistive health technologies are the lack of research in innovation translation, issues with interoperability and usability of assistive health technologies, and the lack of comparative studies across sites and systems [45].

Approximately, 33% of off-the-shelf assistive technologies have been abandoned after being used for 12 months [46]. According to a report by Plaza et al [47], one significant inhibitor for the elderly’s adoption of mobile apps is the ill-designed user interface. As they age, they inevitably experience physical and cognitive capabilities decline. This requires an even more user-friendly interface to encourage their willingness and usage of a technology product.

In order to obtain higher acceptance of technology among the elderly, and thus achieve its potential benefits, it is important to conduct a series of studies or consulting sessions to understand their needs, preferences, and desires for a product [48]. The elderly’s involvement should not be limited to the requirements elicitation stage but all other stages as well (eg, product design, development, and testing).

Some studies have recommended the use of iterative design methods for the designing of mobile apps [49,50]. The iterative design can be defined as a product design process in which the test and evaluation of the deployed product are performed in every stage of design to remove major usability flaws before the product is launched [51].

This study employed the iterative approach to design and develop a digital photo frame–based well-being monitoring system. The approach included three stages: a prototype development, a contextual assessment, and a case study evaluation (across India and Australia). It assured a solid and continuous engagement between the researchers and various stakeholders (the elderly, family members, caregivers, and health care professionals).

The next section presents the three-stage iterative methodology for the design and implementation of a tablet-based well-being check system that uses the “Silvercare” model for its deployment. The study was backed up by ethics approvals in Swinburne University of Technology (Phase 1), the University of New South Wales -Australia (Phases 2 and 3), and the University of Sunshine Coast (Phase 3).

### Phase 1: Initial System Development

**Figure 1:**
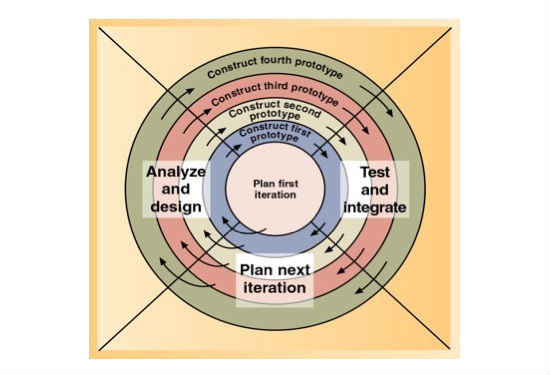
The spiral development model based on agile methodology [51].

A cooperative (consulting with elderly users and service providers) development model was adopted, using an agile (iterative) methodology as shown in Figure 1 [51]. The analysis and design were carried out by a multidisciplinary team (with specializations in public health, aged care, and ICT). The testing was done with elderly users and also with aged care service providers at each stage.

Initial requirements came from some existing elderly alert systems, such as the Mount Eliza Personalised Alert Control System in Victoria, Australia. There were two common features in these systems: (1) a notification of emergency situation (eg, the elderly was able to activate an emergency alert by pressing a button on the pendant) and (2) a well-being check. On a daily basis, the elderly notified their health condition to their trusted service providers by clicking a button in the application window. If no “well-being notification” is sent within a certain period of time, the service provider would contact the elderly to ensure their safety and well-being [52].

Many users of these systems were reluctant to use them. They felt uncomfortable with the integration of new technologies into their daily routine activities. If they did not wear a pendant during, for example, an emergency, they could not receive immediate support from others as required. The findings of this stage have been published in [53].

#### Problems With the Current System

Researchers from Swinburne University of Technology conducted an ethnographic study to identify a design solution for an emergency system based on psychological needs of the end users. In this study, 3 groups of 4 people each (a total of 12) were recruited: (1) a group of senior citizens aged between 85 and 91 years, who had an emergency response system in place; (2) a group of family members whose relatives had experience with the personal alarm system; and (3) a group of senior citizens aged between 66 and 79 years, who had never used the personal alarm system before [54].

The elderly reported that the use of a personal alarm system was not part of their daily routine and its inclusion had an impact on their activities in an unwanted manner, such as follow-up calls from service providers every time when they forgot to press the well-being check button. They believed that there was no social context involved when pressing the well-being check button and the system failed to send the notifications to their family. This led to the need for designing and deploying a new system since the existing personal alarm systems had failed to fulfill the emotional needs of their users [54].

#### The Development of a Well-Being Monitoring Prototype

To address the issues mentioned above, the Smart Services CRC, a software development organization, developed a digital photo frame–based well-being monitoring prototype. Instead of sending a one-way signal or a notification, the prototype allowed the elderly to stay in touch with their family by sharing photos or informing their current health condition through a two-way message exchange. The prototype had two different applications: a mobile app for researchers and a digital photo frame for the elderly. The digital photo frame worked in any Web browsers of computers or tablets (such as iPad).

**Figure 2:**
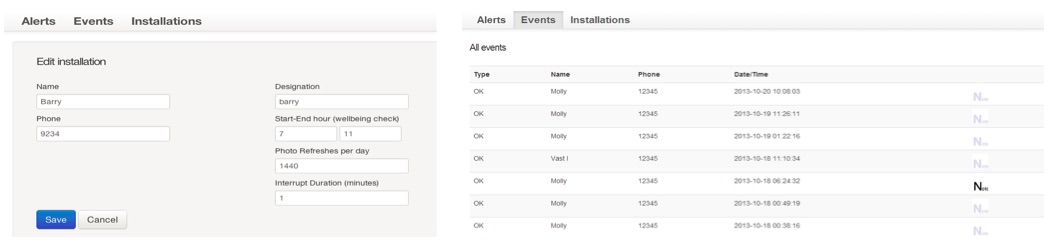
Administrator’s section – alert, event and installation menus.

The mobile app for researchers had several menus, including alert, event, and installation (as shown in Figure 2). They appeared on the left side of the browser window. The installation menu enabled the researchers to add new users into the system. The events menu displayed all recent activities from all users. When the list of events was updated with the latest activity of a particular user, the previous events from other users were placed in chronological order based on the date and time of the latest activity. The alerts menu allowed researchers to monitor any alerts sent by users. There were two types of alerts: investigation and investigation cancel. The “investigation” alert would be triggered if the user had not checked in for a period of time. The “investigation cancel” alert would be triggered if the user had pressed the OK button during the investigation period. Whenever an alarm was triggered, researchers had the responsibility to contact the users directly or to notify their family and/or service providers.

**Figure 3:**
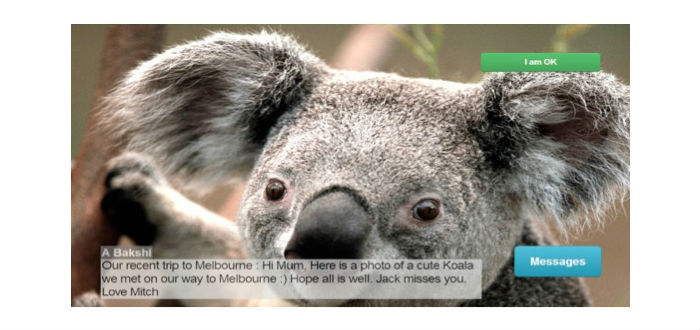
Photo frame user interface.

At the user side, the digital photo frame would display images and messages sent by their family (as shown in Figure 3). Every image had a caption explaining the content of the picture and displayed the alias or sender’s email address. A swipe able carousel-like gallery was also available for displaying multiple images.

#### Testing

The testing was conducted between September and October 2013. It was done with 12 elderly participants aged between 69 and 92 years and residing in Melbourne, Victoria. The results showed that the elderly preferred a more indirect method (eg, to use the digital photo frame) instead of existing emergency alert systems. The elderly agreed that this prototype could be seamlessly integrated with their daily routine and enabled them to maintain social contact with their family [54]. Also, the prototype was demonstrated to a large aged care service provider in Australia. They deemed that the system was not suitable for trial and deployment due to the absence of many required features from their perspective (eg, lack of basic security, lack of voice interface for the elderly, and small fonts)

### Phase 2: Contextual Assessment

**Figure 4:**
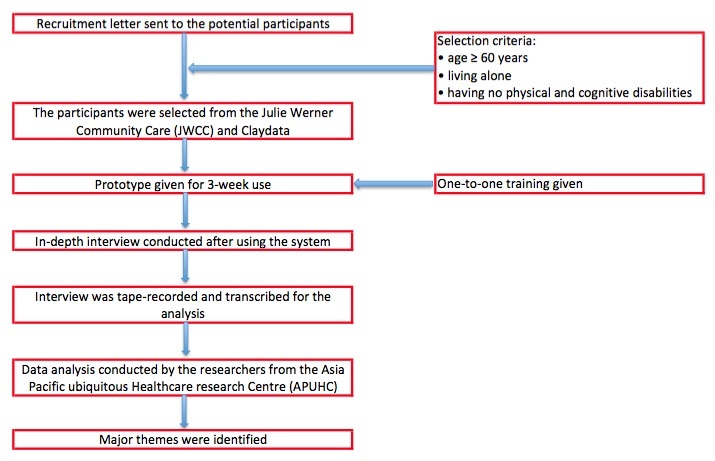
Steps undertaken in a field trial.

On the basis of the collected feedback, the technical team modified the system between November and December 2013. The system added voice-based interfaces and access control, and also enlarged the font size. The users’ perception of the system's usability was evaluated in December 2013. The evaluation process was performed from a health/aged care service provider’s perspective and the elderly’s perspective (Figure 4).

#### Stage 1: Assessment From Service Providers’ Perspectives

A total of 4 participants were selected from residential and community care services. Inclusion criteria included: (1) those directly involved in providing aged care services to the elderly and (2) those who had been working part-time or full-time at the organization for more than one year. The participants had various professional backgrounds, such as business, ICT, and nursing.

Video tutorials on how to use the system were initially provided to the participants. On the completion of the training, they were given access to the prototype and were allowed to try it out for 3 weeks. Subsequently, interviews with open-ended questions were conducted to investigate their perception on the usability and acceptance of the prototype.

The participants appreciated the idea of using photos and two-way messages to socially engage with the elderly and keep their well-being checked. For the ease of use, they were positive. During the discussion, they also suggested the areas with potential for improvement. All suggestions are discussed below.

##### Functional Requirements

Additional functional requirements were collected for possible future development: (1) to allow the elderly to send messages to their service provider if they feel unwell or require immediate help or assistance, (2) to enable service providers to monitor the frequency of communication between the elderly and their family, (3) to send the elderly reminders for medication or meals, and (4) to add an entertainment feature (like games and videos) that would help engage with the user and also improve their cognitive level.

##### Security

At the time of data collection, the app was previewed in a Web browser and had open access to the database, which might lead to a massive loophole for privacy invasion. One suggestion was that the user must enter their username and password to regain access to the system.

##### Ease of Use

In addition to technical issues, one felt that it would be difficult to use for elderly people living with chronic diseases (eg, dementia or visual impairment).

##### Maintainability

It was observed that ongoing support would be costly. For the elderly, on-site support instead of remote support would always be required due to their low level of computer literacy. It was deemed to be critical to design and implement a robust and sustainable support strategy.

#### Stage 2: Assessment From Elderly Users’ Perspectives

A total of 8 elderly people were selected from one community care organization (Table 1). Inclusion criteria included: (1) those aged 60 years and above, (2) those living alone, and (3) those eligible for the low level of care, as per Aged Care Assessment Team assessment. Of the total, 1 participant withdrew from the trial due to health problem. After using the prototype for 3 weeks, the participants were invited for in-depth interviews to examine their perceptions on its usability and acceptance.

##### Attitude

All participants had a positive attitude toward the system and they would like to continue using this system in the future.

##### Design of Interface

Some elders might have forgotten to press “I am OK” due to age-related memory loss and dementia. It would be useful to have another option to automatically generate an “OK” message when the user touches any part of the tablet screen. This feature would enable the service provider to continuously monitor the elderly’s current condition.

Another comment was that pop-up reminders for reminding were too small. One recommendation was to provide a sound alert feature that would notify the elderly when a new message was received or when the medication needed to be taken.

A few who received many photos from their family had difficulties in identifying new photos from old ones. Difficulties were always found in searching for particular photos since the system was set to automatically scroll down the photos. Some recommendations included the use of timestamp when displaying frames, the Trash Can feature for deleting old photos or multiple duplicate photos, and a scroll feature that enables the user to manually scroll through the photos.

##### Ease of Learning

All participants used this system regularly, except one, because of her sickness. They were able to independently operate basic functions like pressing green or red buttons on the screen. However, they were struggling to send messages or to play Web-based games.

The researchers also noticed that most elderly people preferred face-to-face learning environment to the user manual. This might be caused by their low technology skills and unfamiliarity with built-in help functions.

Language barriers had a profound impact on the elderly’s learning process. For those with English as a second language, their children attended all training sessions. Participants were more comfortable with training given in their mother tongue.

##### Ease of Use

In this trial, 5 participants used an iPad and the other 2 used an Android tablet. The latter faced more technical difficulties and were less happy with the size of the screen in comparison with the former. The Android tablet had a shorter battery life and a more sensitive touch screen, which caused more incidents of websites closing accidentally. In addition, because of their unfamiliarity with touch screen devices, the elderly were anxious in using all features of the system at the beginning of the trial.

All participants found it difficult to open the prototype page when they accidently closed the Web browser. Even after training, they were not able to open their account page on the screen by themselves. Each requested easier ways to navigate through their account. A shortcut could be created to the home screen to avoid multiple steps or a pop-up alert like “Are you sure you want to close this program—Yes or No” when a Web browser was about to be closed. However, the number of incidents was reduced after 1 week, as the elderly became more familiar with the system.

##### Perception of Usefulness

Participants agreed that their social connections with their family were significantly improved by using this system. It was also revealed that the ongoing involvement and motivation of the family positively affected their willingness to use such a system.

In addition to personalized communication, many participants found pop-up messages for medication reminders useful **.** One service provider suggested its integration with medication management systems so that care workers would not always be required for the administration of medication to the elderly.

A few participants questioned the usability of the system in the monitoring of well-being. They were looking for a system with an emergency alert feature. For instance, for fall incidents, immediate medical intervention might be required. It is important to mention that this well-being check system has been designed to support the elderly’s independent living and not for an emergency alert.

**Table 1:** The demographic profile of elderly participants in phase 2.

Demography		Number of participants (N=7)
**Age (in years)**		
	60-70 71-80 Above 80	4 2 1
**Gender**		
	Male Female	1 6
**Level of education**		
	Primary school Secondary school Tertiary school	1 3 3

Overall, digital photo frames have a greater chance of being accepted by the elderly living in residential settings [55]. The digital photo frame is generally easier to obtain and this technology merged with a familiar object, only software modifications are needed when upgrading the system and the process is much simpler when compared with other technologies that required hardware modifications [55].

### Phase 3: Case Study Evaluation

The third stage was to examine users’ experiences in using the “well-being communication” system and their attitudes toward the technology in different economic and cultural settings. The comparative case studies were conducted in 2 countries, India and Australia, between September and December 2015. The Indian trial was conducted by the Kalyani Institute for Study, Planning and Action for Rural Change (KINSPARC, a nongovernmental organization that provides aged care services to Indian community) and the Australian trial was conducted by the University of Sunshine Coast and members of the University of the Third Age, located in Sunshine Coast.

The Australian study recruited 30 participants, 27 users and 3 peer tutors. In India, there were 20 participants. The average age of participants was 70.8 years. There were 31 females (66%, 31/47) and 16 males (34%, 16/47). Of the 20 participants in India, 8 had never had any experience of using an electronic table, computer, or mobile phone.

Inclusion criteria for the elderly included: (1) those aged 65 years and over, (2) those living alone, and (3) those who had no record of cognitive impairments and blindness. Inclusion criteria for the care coordinator staff included: (1) those who had the time and would be willing to help the elderly in their area by physically visiting them at least once in a week, (2) those who were comfortable with the use of mobile phones, and (3) those supported by pension (did not need any financial support from this project). The demographic profile of participants in phase 3 is summarized in Table 2. There were 27 participants from Australia (Sunshine Coast) and 20 from India (Kalyani).

In order to overcome the problem of deployment and maintainability, this comparative study adopted the concept of “Silvercare,” which involves young retired people as coordinators who support about ten elderly people in their geographical vicinity [56].

In these comparative studies, Dropbox had been selected for sharing and storing photographs safely, while Skype for video conference calls. The user acceptance of the “well-being communication” system was evaluated with 7 variables (anxiety, attitude, facilitating condition, intention to use the system, self-efficacy, social influence, and usefulness).

The effect of potential moderators (like age, gender, and experience) on the independent variable was also investigated in this study. After using the “well-being communication” system for 2 months, each participant was asked to fill out 2 surveys. They were developed based on the unified theory of acceptance and use of technology model to collect quantitative data [57]. The interview guide used at Stage 2 was reused to collect qualitative data. This phase of the evaluation has been reported in detail in [58].

**Table 2:** The demographic profile of elderly participants in a comparative evaluation [58].

Demography		Total (n=47), n (%)	Australia (n=27), n (%)	India (n=20), n (%)
**Age (in years)**				
	60-70	23 (49)	11 (41)	12 (60)
	71-80	17 (36)	12 (44)	5 (25)
	Above 80	7 (15)	4 (15)	3 (15)
**Gender**				
	Male	16 (34)	9 (33)	7 (35)
	Female	31 (66)	18 (67)	13 (65)
**Level of education**				
	High school/GED	8 (17)	5 (19)	3 (15)
	Diploma course	6 (13)	6 (22)	0 (0)
	Bachelor’s degree	20 (43)	8 (30)	12 (60)
	Master’s degree	9 (19)	5 (18)	4 (20)
	Doctoral degree	1 (2)	0 (0)	1 (5)
	Vocational/ technical	3 (6)	3 (11)	0 (0)
**Previous technological experience**				
	Used an electronic tablet, mobile phone or computer before	39 (83)	27 (100)	12 (60)

Anxiety: Most participants confirmed that they had not been anxious when using those applications (Australia=85.2%, India=60.0%). Some felt intimidated (Australia=3.7%, India=20.0%) and even feared of making mistakes (Australia=11.1%, India=25.0%).

Attitude: All participants had a positive attitude toward the applications. The obtained results accorded well with findings from previous study [59]. For example, the Australian participants believed that the applications were good ideas (Skype Australia=88.9%, Dropbox Australia=85.2%), easy to operate on the tablet (Skype Australia=62.9%, Dropbox Australia=59.2%), enjoyable (Skype Australia=66.6%, Dropbox Australia=66.6%), and enabled active communication (Skype Australia=74.2%, Dropbox Australia=62.9%).

Facilitating condition: From the observation, the participants who had experience with computers or mobile phones were able to use those applications without any difficulties (Australia=74.1%, India=80.0%). They also agreed that those applications were well fitted in their current lifestyle (Australia=66.6%, India=80.0%).

Intention to use the system: A majority of users agreed or strongly agreed that the applications were useful (Skype Australia=62.9%, Dropbox Australia=37.0%, India=95.0%) and indicated that using them improved their communication (Skype Australia=55.9%, Dropbox Australia=51.8%, India=80.0%). Some admitted that these applications were able to increase the frequency of communication with their family.

Self-efficacy: Despite the fact that most participants were able to use those applications (Skype Australia=70.4%, Dropbox Australia=66.7%, India=45.0%), they preferred to seek help from professional sources (Skype Australia=81.5%, Dropbox Australia=70.0%, India=55.0%) or the Web (Skype Australia=44.4%, India=65.0%).

Social influence: It was shown that the family’s ongoing involvement and motivation positively affected their willingness to use such applications (Skype Australia=59.2%, Dropbox Australia=55.5%, India=95.0%).

Usefulness: Findings also revealed that those applications were easy to use (Skype Australia=74.2%, Dropbox Australia=62.9%, India=80.0%) and that it did not take much time to learn (Skype Australia=66.6%, Dropbox Australia=62.9%, India=75.0%).

Overall, the use of mobile apps on tablets has been quite useful in both India and Australia, though a small number of participants have not accepted the technology. It was heartening to see some of the elderly people latching onto this communication mechanism wholeheartedly. The detailed explanation of this stage has been published in [58]. The results cannot be generalized for a population in a large country with diverse population as in India in view of the small sample size and study conducted in one place. However, the project has successfully tested the iterative methodology for the design of IT systems for the care of the elderly.

## Discussion

This paper presented the experience of this multiphase project on the development of tablet-based well-being check for the elderly. The paper presented a new three-phase iterative methodology for the cooperative system design and implementation. The three phases involved were:

Phase 1: The development of a prototype for a tablet-based well-being check using an agile design methodology. It allows the elderly to, for example, share photos or inform their current health condition through a two-way message exchange.

Phase 2: A contextual assessment of this technology of the elderly users in collaboration with aged care providers and family members of the elderly. The areas (eg, privacy features) for improvement were identified.

Phase 3: A case study evaluation of the tablet-based well-being check in the homes of the elderly across India and Australia. Between participants from the two countries, the results highlighted their different levels of familiarity with technology. In all cases, their family support was crucial to their willingness to use the technology.

As stated in the Methods section, there were changes made in the system after every phase based on the feedback of users (elderly, family members, and aged care providers) on functionality, security, usability, and maintainability. The same methodology can be used for the development of various IT systems and services for the elderly.

We now summarize below the lessons learned from this project based on the three main contributions:

First, development and testing of digital photo frames to facilitate communication between the elderly, family members, and the carers. The seniors said that the system had become a part of their daily routine. They were looking for the digital frame when they woke up in the morning and also used it occasionally during the day. On the other side, the community service provider agreed on the potential benefits of the system, especially for monitoring of well-being of the elderly. They are now demanding more sophisticated forms of user interface and also security management applications.

Second, this research illustrated a new iterative design methodology (three phases) to design, test, and evaluate the tablet-based well-being check system for the elderly. This methodology evolved while we were executing the project. We initially started the design based on traditional system development models. However, it became clear while carrying out the field study that multiple phases would be required.

Third, implementation and testing of the pragmatic “Silvercare” model. We had to use this innovative model to effectively educate, train, and support the elderly in the use of the tablet-based system. That way we were able to incorporate the peculiar support needs (different from those for younger users) for IT systems for the elderly. However, this “Silvercare” model is generic enough to be used for a range of aged care services in different parts of the world.

We hope this paper will lead to more research in the three areas of our contribution and possibly more new research on this important subject in view of the aging population all over the world.

## Abbreviations

ICT: information and communications technology
IT: information technology
